# Emergomycosis, an Emerging Thermally Dimorphic Fungal Infection: A Systematic Review

**DOI:** 10.3390/jof9101039

**Published:** 2023-10-23

**Authors:** Kalaiselvi Vinayagamoorthy, Dinesh Reddy Gangavaram, Anna Skiada, Hariprasath Prakash

**Affiliations:** 1 Centre for Public Health (U.I.E.A.S.T), Panjab University, Chandigarh 160014, Punjab, India; drkalai1626@gmail.com; 2 Department of Dermatology, Venereology and Leprosy, PES Institute of Medical Sciences & Research, Kuppam 517425, Andhra Pradesh, India; gdinesh6887@gmail.com; 3 First Department of Medicine, Laiko Hospital, National and Kapodistrian University of Athens, 11527 Athens, Greece; askiada@yahoo.com; 4 Department of Microbiology, PES Institute of Medical Sciences & Research, Kuppam 517425, Andhra Pradesh, India

**Keywords:** *Emergomyces*, emergomycosis, *Emergomyces africanus*, dimorphic fungi, endemic mycosis

## Abstract

Emergomycosis is an endemic mycosis caused by the *Emergomyces* species. Infections due to this agent have been reported globally. Hence, the present systematic review on *Emergomyces* infections was conducted to study the disease epidemiology, underlying diseases and risk factors, causative agents, and treatment and outcome. The MEDLINE, Scopus, Embase, and Web of Science databases were searched systematically with appropriate keywords from January 1990 to October 2022. A total of 77 cases of emergomycosis were included in the analysis. Emergomycosis was most commonly seen in patients with human immunodeficiency virus (HIV) infection (n = 61, 79.2%) and HIV-uninfected patients with or without other comorbidities (n = 16, 20.8%). The underlying disease and risk factors significantly associated with emergomycosis in the HIV-infected patients were CD4+ T-cell counts less than 100 cells/mm^3^ (n = 55, 90.2%), anaemia (n = 30, 49.2%), and thrombocytopenia (n = 17, 27.9%), whereas in the HIV-uninfected patients, treatment with immunosuppressive drugs (n = 10, 62.5%), renal disease (n = 8, 50%), transplant recipients (n = 6, 37.5%), and diabetes mellitus (n = 4, 25%) were the significant risk factors associated with emergomycosis. *Emergomyces africanus* (n = 55, 71.4%) is the most common causative agent, followed by *E. pasteurianus* (n = 9, 11.7%) and *E. canadensis* (n = 5, 6.5%). *E. africanus* was most often isolated from HIV-infected patients (n = 54, 98.2%), whereas *E. pasteurianus* was most common in HIV-uninfected patients (n = 5, 55.6%). The all-cause mortality rate of the total cohort is 42.9%. No significant variation in the mortality rate is observed between the HIV-infected patients (n = 28, 36.4%) and the HIV-uninfected patients (n = 5, 6.5%). In conclusion, with an increase in the immunosuppressed population across the globe in addition to HIV infection, the case burden of emergomycosis may increase in the future. Hence, clinicians and mycologists should be vigilant and clinically suspicious of emergomycosis, which helps in early diagnosis and initiation of antifungal treatment to prevent disease mortality.

## 1. Introduction

Emergomycosis is an emerging thermally dimorphic fungal infection caused by the genus *Emergomyces* (formerly placed under the genus *Emmonsia*), belonging to the order *Onygenales* and family *Ajellomycetaceae* [1,2]. The taxonomy of fungal species in the family *Ajellomycetaceae* is rapidly evolving using a phylogenetic or phylogenomic approach, and multiple new species and genera have been described [1,2]. Multi-gene phylogenetic analyses on *Emmonsia* and *Emmonsia*-like fungal species revealed that the genus is polyphyletic [1,2]. Further, phylogenetic studies showed that *Emmonsia* species were clustered closely with the genus *Emergomyces* and *Blastomyces* [1,2,3]. Hence, the species described previously under *Emmonsia* were taxonomically repositioned, either in the genus *Emergomyces* or *Blastomyces* [1,2,3]. These recent phylogenetic changes may render the genus *Emmonsia* obsolete. Based on molecular phylogenetic analyses, seven species are currently placed in the genus *Emergomyces*, namely *E. africanus*, *E. canadensis*, *E. crescens*, *E. europaeus*, *E. orientalis*, *E. pasteurianus*, and *E. sola* [1,2,3]. Except for *E. sola* (non-pathogenic soil saprobe, not associated with human infections), the other pathogenic *Emergomyces* species are known to cause invasive human diseases [4].

The *Emergomyces* species is a soil saprobe, and humans may acquire the infection by inhaling the contaminated spores from the environment. Infections due to *Emergomyces* species are rising globally, and the cases are being reported from Africa, Asia, Europe, North America, and South America [4,5]. However, a high case burden has been seen in Africa, specifically in South Africa [6,7]. Further, soil samples analysed using molecular techniques from South Africa reported a 30% positivity rate for *E. africanus*, a primary causative agent of human-disseminated emergomycosis in Africa [8]. Similarly, *E. crescens*, causing granulomatous pulmonary adiaspiromycosis in humans and rodents, has been isolated from soils and rodent tissue samples [9]. The ecological niche of other pathogenic *Emergomyces* species is largely unknown. The significant risk factors associated with emergomycosis were human immunodeficiency virus (HIV) infection, transplant recipients, and malignancies [4,5]. However, occasionally, infections are reported in apparently healthy immunocompetent hosts [4]. The disease manifests as skin lesions, pulmonary infection, fungemia, or severe disseminated disease. Most patients with *Emergomyces* infections are immunocompromised; early diagnosis is essential to initiate appropriate antifungal therapy. The mortality rate in emergomycosis patients ranges from 48 to 51% [6,10]. With this background, the present systematic review was conducted to understand the disease epidemiology, underlying disease and risk factors, and treatment and outcome associated with emergomycosis.

## 2. Methods

### 2.1. Study Design, Selection, and Data Extraction

**Figure 1:**
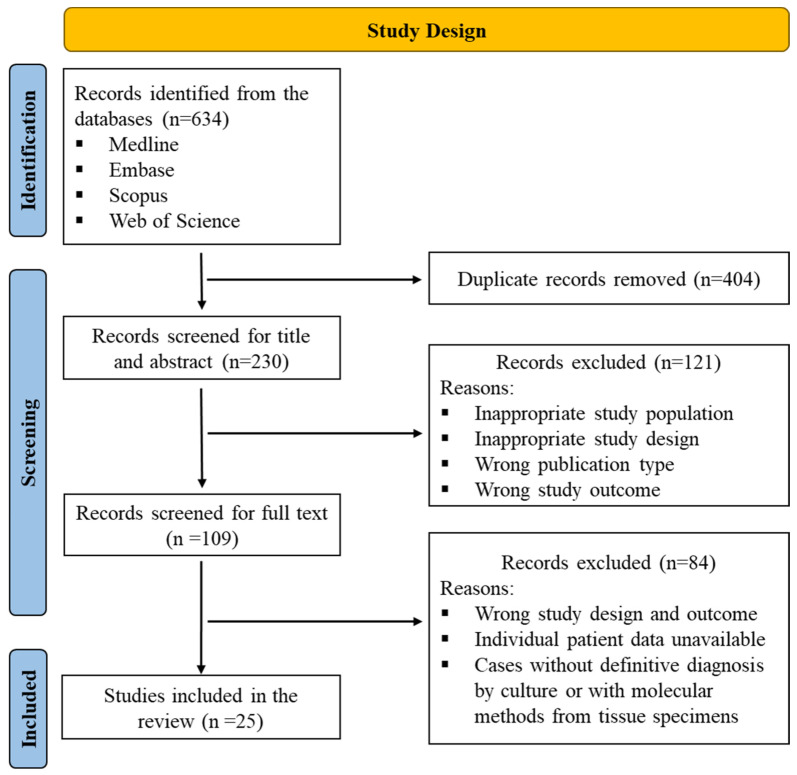
PRISMA flowchart depicting the study design and process.

The systematic review was conducted per PRISMA guidelines and has been registered with PROSPERO (registration number: CRD42023375274). Studies on *Emergomyces* infections published between January 1990 and October 2022 were searched from the following databases: Medline, Embase, Scopus, and Web of Science, along with back-references of the published literature. The citations of the studies that matched the search strategy were retrieved from these databases and uploaded to the Rayyan QCRI software [11]. Duplicates were removed, then the title and abstract of the articles were screened for inclusion by two independent reviewers (K.S.V and H.P.P), and discrepancies were fixed via discussion and consensus with the third reviewer (A.S.). Further, full texts were screened for inclusion of the studies in the review (K.S.V and H.P.P), and the studies that failed to fulfil the inclusion criteria were excluded from the analysis. A detailed study description is given in Figure 1.

Only proven cases of *Emergomyces* infections, either with culture or with molecular methods from tissue specimens (definitive diagnosis of emergomycosis), were included in the analysis. Studies with proven *Emergomyces* infections published in English texts, such as case series, case reports, prospective and retrospective studies, and conference papers with complete case details, were included in the review. The data from the included studies, such as country of the study, number of *Emergomyces* cases reported, patient’s age, sex, underlying diseases and risk factors, mode of diagnosis, causative agents, antifungal treatment, and outcome of the disease (i.e., mortality) were extracted (K.S.V and D.R.G). Further, the taxonomy of *Emergomyces* was revised recently; hence, we contacted the authors to confirm the species identification (only when the speciation of the isolates was not clearly defined). Further, this review excluded narrative and mini reviews, systematic and meta-analysis studies, editorials, and non-English literature.

### 2.2. Risk of Bias Assessment

The included articles were assessed for risk of bias using a modification of the Joanna Briggs Institute (JBI) tool for the case series [12,13]. The articles were categorised as having a low, high, or unclear risk of bias under the following domains: clear inclusion criteria, a valid identification method, clear reporting of the demographic information, clinical parameters, outcomes, and the presenting site(s)/clinic(s) of demographic information.

### 2.3. Statistical Analysis

The clinical details extracted from emergomycosis cases, such as underlying diseases and risk factors, mode of diagnosis, causative agents, treatment, and the disease outcome between the groups (HIV-infected and HIV-uninfected patients), were compared. We performed the χ^2^ test (Fisher’s exact test) using SPSS version 22 to draw the statistical inference. A two-sided *p*-value < 0.05 was considered significant.

## 3. Results

A total of 25 studies that fulfilled the inclusion criteria were analysed, and the risk of bias was evaluated [10,14,15,16,17,18,19,20,21,22,23,24,25,26,27,28,29,30,31,32,33,34,35,36,37,38]. Three studies had a high risk of bias in one of the domains assessed (in two studies, clinical findings were not described [30,38]); and one study had an unclear risk of bias, failing to document the disease outcome properly [33]). Of these studies, a total of 77 proven cases of emergomycosis either with culture (n = 72, 93.5%) or with molecular diagnosis from tissue specimens (n = 5, 6.5%) were included in the study. By geographic distribution, most cases were from Africa (n = 57, 74%), Asia (n = 7, 9.1%), North America (n = 7, 9.1%), Europe (n = 5, 6.5%), and South America (n = 1, 1.3%). Adults were most commonly affected (n = 75, 98.7%) with one paediatric case. The age group of the patients ranged between 03 and 80 years. The mean age of the patients was 39.25 years (standard deviation ± 13.28). Males were more commonly affected (n = 47, 61%). The male and female ratio is 1.57:1. The clinical details, such as clinical manifestations, underlying diseases, and risk factors, are summarised in Table 1 and Table S1.

For comparative analysis based on the underlying disease and risk factors, we categorised the patients into two groups: (a) HIV-infected and (b) HIV-uninfected patients with or without other comorbidities. Of the 77 cases analysed, most were seen in patients with HIV infection (n = 61, 79.2%). Emergomycosis was more commonly seen in HIV-infected patients with CD4+ T-cell counts less than 100 cells/mm^3^ (n = 55, 90.2%, *p* < 0.0001). Further, anaemia (n = 30, 49.2%, *p* = 0.010) and thrombocytopenia (n = 17, 27.9%, *p* = 0.016) were the significant risk factors in the HIV group. Whereas in the HIV-uninfected patients (n = 16, 20.8%), the significant risk factors associated with emergomycosis were patients on immunosuppressive drugs (n = 10, 62.5%, *p* < 0.0001), renal disease (n = 8, 50%, *p* < 0.0001), transplant recipients (n = 6, 37.5%, *p* < 0.0001), and diabetes mellitus (n = 4, 25%, *p* = 0.006). Further, in the HIV-uninfected patients, four were apparently healthy, and no risk factors were ascertained (n = 4, 25%, *p* = 0.001). The other risk factors such as opportunistic infections were most common in the HIV-infected (n = 26, 33.8%), compared to the HIV-uninfected patients (n = 3, 3.9%, *p* = 0.092). Table S2 summarises the opportunistic infections in the emergomycosis patients.

The disease manifested as skin lesions in most of the HIV patients (n = 58, 95.1%), compared to the HIV-uninfected patients (n = 7, 43.8%, *p* < 0.0001) (Table S3). A subset analysis of HIV-infected patients did not identify any other significant risk factors, such as patients not receiving anti-retroviral therapy (ART) or ART-defaulted patients before the appearance of skin lesions. The lesions primarily appeared as papules (n = 32, 41.6%), nodules (n = 17, 22.1%), crust (n = 15, 19.5%), and plaques (n = 11, 14.3%) in both groups (Table S3), and the appearances/clinical descriptions of the skin lesions were not statistically significant between HIV-infected and HIV-uninfected patients (Table S3). Other than skin lesions, the disease manifested as pulmonary disease in HIV-uninfected patients (n = 12, 75%, *p* = 0.000l). Table S4 describes the chest and abdominal radiological findings in the emergomycosis patients. Abnormal chest radiological findings such as lung opacities and lung infiltrates were more common in the HIV-uninfected patients (n = 14, 87.5%) compared to the HIV-infected patients (n = 32, 52.5%, *p* = 0.011). Contrastingly, in the HIV-infected patients, the dissemination of the disease is seen, and the causative agents are isolated from blood (n = 23, 37.7%, *p* = 0.074) and bone marrow specimens (n = 14, 23%, *p* = 0.034) more frequently (Table 2).

The specimens and diagnostic methods used in the emergomycosis detection are described in Table 2. Most of the cases were diagnosed with histology and culture (n = 50, 64.9%), culture alone (n = 22, 28.6%), and histology and molecular detection from tissue specimens (n = 5, 6.5%). Bone marrow specimens in HIV patients (n = 14, 23%, *p* = 0.034) and the respiratory specimens (n = 12, 75%, *p* = 0.0001) in the HIV-uninfected patients showed higher isolation rate for *Emergomyces* species. For the culture-proven cases, due to the taxonomy reclassification of *Emergomyces* species, we contacted the authors of the study (only when proper speciation is not mentioned in the article) to confirm the species identification. The majority of the cases in this review were due to *E. africanus* (n = 55, 71.4%), followed by *E. pasteurianus* (n = 9, 11.7%), *E. canadensis* (n = 5, 6.5%), *E. crescens* (n = 2, 2.6%), *E. orientalis* (n = 2, 2.6%), and *E. europaeus* (n = 1, 1.3%). However, in three cases, the species identification of *Emergomyces* was not available. *Emergomyces africanus* was significantly isolated from the HIV-infected patients (n = 54, 98.2%, *p* = 0.0001). In comparison, *E. pasteurianus* (n = 5, 55.6%, *p* = 0.016) was more commonly isolated from the HIV-uninfected patients.

Treatment and outcome of an *Emergomyces* infection are summarised in Table 1 and Table S5. The all-cause mortality of the total cohort was 42.9% (n = 33). Antifungal therapy was initiated in 64 (83.1%) patients, and 9 (11.7%) patients did not receive any antifungal drugs; the mortality rates in these groups were 34.4% and 88.9%, respectively (*p* = 0.003). Most patients received combination therapy of amphotericin B and azoles (n = 34, 44.2%), and in this group, the mortality rate was 23.5% (n = 8, *p* = 0.003). Further, the subset analysis showed that amphotericin B and itraconazole were given to 24 patients, and in these patients, the mortality rate decreased to 20.8% (n = 5, *p* = 0.012). Further, we assessed the mortality rate between the HIV-infected (n = 28, 45.9%) and the HIV-uninfected patients (n = 5, 31.3%, *p* = 0.397), where no significant difference was observed.

## 4. Discussion

The present systematic review discusses the underlying diseases and risk factors, causative agents, treatment, and disease outcome in emergomycosis cases. This review identified 77 proven emergomycosis cases either with culture or with direct detection using molecular methods from tissue specimens that were analysed from January 1990 to October 2022. The causative agents of emergomycosis infections were *E. africanus* (n = 55, 71.4%), *E. pasteurianus* (n = 9, 11.7%), and *E. canadensis* (n = 5, 6.5%). Most emergomycosis cases were seen in HIV-infected patients (n = 61, 79.2%). The underlying diseases and risk factors for emergomycosis in the HIV-infected patients were CD4+ T-cell counts (<100 cells/mm^3^), anaemia, and thrombocytopenia (Table 1). HIV-uninfected patients on immunosuppressive drug therapy, transplant recipients, renal diseases, and diabetes mellitus were at significant risk for emergomycosis. Further, few cases of emergomycosis have been reported in apparently healthy immunocompetent hosts (Table 1).

Most of the emergomycosis cases described in this review are reported from Africa (n = 57, 74%), specifically high burden is seen in South Africa (n = 56, 98.2%). *E. africanus* is the most common agent causing emergomycosis in South Africa (n = 55, 96.5%). The reason for the highest case burden in South Africa is unknown. The possible reason for the increased case burden of emergomycosis in South Africa may be attributed to the high prevalence of the HIV-infected population and non-adherence to anti-retroviral therapy, high clinical awareness, or high spore burden of *Emergomyces* species in South African soils [4,5,7]. An environmental study from South Africa showed that 30% of the analysed soil samples contained *E. africanus* [8]. It is presumed that patients acquire the infection via inhalation of the spores from the environment. A study from South Africa analysed the air propagules using quantitative PCR, and the study reported that 10% of the samples analysed contained *E. africanus* [39]. These findings showed that, possibly, the high spore burden due to *Emergomyces* spores in the environment and the host factors (immunocompromised conditions such as HIV, malignancies, and solid organ transplant recipients) may be the prime factors contributing to the disease burden. Further, other than *E. africanus*, infections due to newly described *Emergomyces* species are reported across continents. However, the ecological niche of those *Emergomyces* species has yet to be known. In contrast to *E. africanus*, the second most common agent of emergomycosis, *E. pasteurianus* causes infection more often in HIV-uninfected patients (n = 5, 31.3%), compared to HIV-infected patients (n = 4, 6.6%, *p* = 0.016).

In the present review, patients with emergomycosis often presented with skin lesions (n = 65, 84.4%); however, the skin lesions were most common in the HIV patients (75.3%) compared to the HIV-uninfected patients (9.1%) (Table S3). Contrastingly, in the HIV-uninfected patients, emergomycosis presented as respiratory disease; the respiratory specimens yielded a higher isolation rate of *Emergomyces* species in the HIV-uninfected patients (75%) compared to 5% in the HIV-infected patients (*p* = 0.0001). Further, blood (37.7%) and bone marrow (23%, *p* = 0.034) specimens had higher positive culture rates in HIV-infected patients, showing the disseminated nature of the disease in the HIV-infected patients. The risk factors for emergomycosis in HIV-infected patients were CD4+ T-cell counts (<100 cells/mm^3^), anaemia, and thrombocytopenia. In HIV-uninfected patients, treatment with immunosuppressive drugs and transplant recipients were at significant risk for emergomycosis (Table 1). These findings show that the lack of a cell-mediated immune response is the most significant risk factor for emergomycosis.

In the present review, the all-cause mortality rate of emergomycosis was 42.9%. At the same time, the difference in the mortality rate was not statistically significant between the HIV-infected patients (36.4%) compared to 6.5% in the HIV-uninfected patients. Similarly, the other studies documented a mortality rate of 48–51% [6,10]. There are no standard treatment guidelines for the management of emergomycosis. The treatment guidelines devised by the Infectious Diseases Society of America (IDSA) for managing other endemic mycoses, such as histoplasmosis, are currently followed in treating emergomycosis infections [40,41,42]. The treatment and outcome of the disease are described in Table 1 and Table S5. Patients on antifungal therapy had a lesser mortality rate, 34.4%, than those without antifungal treatment (89%, *p* = 0.003). Amphotericin B and triazole combination therapy had a better survival rate at 76.5% (*p* = 0.003). Amphotericin B and itraconazole therapy effectively reduced the mortality rate in patients with emergomycosis (20.8%, *p* = 0.012). Further, triazole alone was used as therapy in 19 (24.7%) patients, and the mortality rate was 36.8% (*p* = 0.602). These findings showed that the combination therapy of amphotericin B and azole specifically itraconazole, may help in the improved clinical outcome of the disease. These findings correlate with the in vitro antifungal susceptibility testing results, as amphotericin B was the most effective drug against the *Emergomyces* species [43,44]. Similarly, itraconazole has low minimum inhibitory concentrations, making it the ideal candidate for step-down therapy following amphotericin B [43,44].

## 5. Conclusions

This review highlights the emergence of new thermal dimorphic fungi, the *Emergomyces* species, and their importance in clinical settings as this agent is associated with high mortality. The disease prevalence of emergomycosis is not known; population-based studies are necessary to estimate the true prevalence/incidence. Patients with HIV, malignancy, and transplant recipients are at increased risk for emergomycosis. In the future, an increase in emergomycosis infections may be seen in patients with defective cell-mediated immunity or with immunosuppressive therapy. Further, emergomycosis is an endemic disease, and a high index of clinical suspicion is needed for early diagnosis and treatment. However, the disease has recently been seen in non-endemic areas, and many new species have been described. Hence, a molecular phylogenetic approach and genomic studies are essential to understanding disease epidemiology. Emergomycosis is associated with high mortality; further, there are no standard treatment guidelines available. Based on the available data, combination therapy with amphotericin B and step-down therapy with itraconazole may help achieve better survival rates. In future, clinical trials or in vivo animal model studies are essential to understand the efficacy of different antifungal agents against the *Emergomyces* species and to design treatment guidelines.

## Figures and Tables

**Table 1:** Clinical details of emergomycosis patients.

Parameters	Total N (%)	HIV-Infected Patients N (%)	HIV-Uninfected Patients N (%)	*p* Value
Total number of patients	77 (100)	61 (79.2)	16 (20.8)	-
Age (in range) ^a^	3–80	3–68	17–80	-
Mean age in years (±SD) ^a^	39.25 (13.28)	36.38 (10.48)	50 (17.12)	-
Paediatric (0–16 years) ^a^	1 (1.3)	1 (1.3)	0	>0.99
Adult (>16 years) ^a^	75 (98.7)	59 (78.7)	16 (21.3)	>0.99
Male and female ratio	1.57:1	1.26:1	4.3:1	-
Male	47 (61)	34 (44.2)	13 (16.9)	0.085
Female	30 (39)	27 (35.1)	03 (3.9)	0.085
Cases reported across continents ^b^				
Africa	57 (74)	56 (72.7)	1 (1.3)	0.0001 *
Asia	7 (9.1)	2 (2.6)	5 (6.5)	0.004 *
North America	7 (9.1)	2 (2.6)	5 (6.5)	0.004 *
Europe	5 (6.5)	1 (1.3)	4 (5.2)	0.006 *
South America	1 (1.3)	0	1 (1.3)	0.208
Underlying diseases and risk factors				
CD4+ T-cell counts (<100 cells/mm^3^)	55 (71.4)	55 (71.4)	0	0.0001 *
Anaemia	32 (41.6)	30 (39)	2 (2.6)	0.01 *
Thrombocytopenia	17 (22.1)	17 (22.1)	0	0.016 *
Immunosuppressive drugs	11 (14.3)	1 (1.3)	10 (13)	0.0001 *
Renal diseases	9 (11.7)	1 (1.3)	8 (10.4)	0.0001 *
Transplant ^c^	7 (9.1)	1 (1.3)	6 (7.8)	0.0001 *
Malignancy ^d^	5 (6.5)	2 (2.6)	3 (3.9)	0.058
Diabetes mellitus	5 (6.5)	1 (1.3)	4 (5.2)	0.006 *
Immunocompetent host	4 (5.2)	0	4 (5.2)	0.001 *
History of past pulmonary tuberculosis	17 (22.1)	17 (22.1)	0	0.016 *
Cholestatic disease	20 (26)	20 (26)	0	0.008 *
Acute kidney injury	9 (11.7)	9 (11.7)	0	0.191
Antifungal Treatment ^e^				
Patients with antifungal treatment	64 (83.1)	50 (64.9)	14 (18.2)	0.740
No antifungal treatment	9 (11.7)	8 (10.4)	1 (1.3)	0.740
Amphotericin B alone	8 (10.4)	7 (9.1)	1 (1.3)	>0.99
Triazole alone	19 (24.7)	12 (15.6)	7 (9.1)	0.058
Amphotericin B and azole combination therapy	34 (44.2)	30 (39)	4 (5.2)	0.098
Amphotericin B, azoles and echinocandins	3 (3.9)	1 (1.3)	2 (2.6)	0.108
The outcome of the disease ^f^				
Survived	44 (57.1)	33 (42.9)	11 (14.3)	0.397
Death	33 (42.9)	28 (36.4)	5 (6.5)	

Note: The values in the table are expressed in N (number) and % (percentage). The percentages in the table were calculated using the total number of patients (N) as the denominator. * The *p* values < 0.05 were considered statistically significant. ^a^ Age was reported in 76 cases. Age was not reported in one case [20]. The mean age (±standard deviation (SD)) was calculated using 76 cases. ^b^ Countries from emergomycosis cases reported were Africa: South Africa (n = 56) and Uganda (n = 1); Asia: China (n = 4), India (n = 2), and Hong Kong (n = 1); North America: Canada (n = 2), the United States of America (n = 1), and not reported (n = 4); Europe: Netherlands (n = 2), France (n = 1), Germany (n = 1), and Spain (n = 1); and South America: Argentina (n = 1). ^c^ Organs transplanted were kidney (n = 6) and liver (n = 1). ^d^ Types of malignancy: B cell chronic lymphocytic leukaemia (n = 1), Hepatocellular carcinoma (n = 1), Kaposi sarcoma (n = 1), Large B Cell non-Hodgkin’s lymphoma (n = 1), and Osteosarcoma (n = 1). ^e^ Antifungal treatment data were available in 73 cases. Four patients did not have treatment data [10,30]. ^f^ One study failed to report disease outcomes (n = 1) clearly. However, based on available data, disease outcomes from this study were included in the survival group [33].

**Table 2:** Diagnostic methods and causative agents of emergomycosis.

Diagnostic Methods	Total N (%)	HIV-Infected Patients N (%)	HIV-Uninfected Patients N (%)	*p* Value
Histology and culture	50 (64.9)	38 (49.4)	12 (15.6)	-
Culture only	22 (28.6)	20 (26)	2 (2.6)	-
Histology and molecular detection from direct clinical specimens	5 (6.5)	3 (3.9)	2 (2.6)	-
Specimens positive via histology ^a^				
Skin biopsy	47 (61)	41 (53.2)	6 (7.8)	0.044 *
Respiratory specimens	12 (15.6)	2 (2.6)	10 (13)	0.0001 *
Bone marrow	3 (3.9)	3 (3.9)	0	>0.99
Liver	2 (2.6)	2 (2.6)	0	>0.99
Blood	1 (1.3)	1 (1.3)	0	>0.99
Clinical specimens positive via culture or molecular methods ^b^				
Skin biopsy	35 (45.5)	30 (39)	5 (6.5)	0.263
Blood	25 (32.5)	23 (29.9)	2 (2.6)	0.074
Respiratory specimens	15 (19.5)	3 (3.9)	12 (15.6)	0.0001 *
Bone marrow	14 (18.2)	14 (18.2)	0	0.034 *
Causative agents ^c^				
*Emergomyces africanus*	55 (71.4)	54 (70.1)	1 (1.3)	0.0001 *
*Emergomyces pasteurianus*	9 (11.7)	4 (5.2)	5 (6.5)	0.016 *
*Emergomyces canadensis*	5 (6.5)	2 (2.6)	3 (3.9)	0.058
*Emergomyces crescens* ^d^	2 (2.6)	0	2 (2.6)	0.041 *
*Emergomyces orientalis*	2 (2.6)	0	2 (2.6)	0.041 *
*Emergomyces europaeus*	1 (1.3)	0	1 (1.3)	0.208
*Emergomyces species* ^d^	3 (3.9)	1 (1.3)	2 (2.6)	0.108

Note: The values in the table are expressed in N (number) and % (percentage). The percentages in the table were calculated using the total number of patients (N) as the denominator. * The *p* values < 0.05 were considered statistically significant. ^a^ Histological positive cases of emergomycosis cases were n = 55. Patients with at least one clinical sample positive for emergomycosis with histological examinations were n = 47 (HIV-infected (n = 35) and HIV-uninfected (n = 12)), and patients with more than one clinical specimen positive for emergomycosis were n = 8 (HIV-infected (n = 6) and HIV-uninfected (n = 2)). ^b^ A total of 77 cases of emergomycosis were identified in this study; of that, culture-proven cases were n = 72, and direct detection from tissue specimens with molecular methods was n = 5. Patients with at least one clinical sample positive for culture were n = 61 (HIV-infected (n = 50) and HIV-uninfected (n = 11)), and patients more than one clinical specimen positive were n = 11 (HIV-infected (n = 8) and HIV-uninfected (n = 3)). Clinical specimens positive for direct detection with molecular methods were skin biopsy (n = 3), bronchoalveolar lavage, tracheal aspirate and lung biopsy (n = 1), and lung tissue (n = 1). ^c^ Based on the data provided by the authors and genes used in the identification of *Emergomyces* from culture and tissue specimens, there were internal transcribed spacer (ITS) region (n = 72), large ribosomal subunit 28S rDNA (n = 60), beta-tubulin (n = 2), small ribosomal subunit 18S rDNA (n = 1), actin (n = 1), and intein PRP8 gene (n = 1). Two studies used molecular methods but failed to report the genes used [24,32]. Schwartz et al. communicated that ITS genes were used initially, followed by 28S rDNA, and further whole genome sequencing was used in the identification of *E. africanus* isolates [10]. ^d.^ Cultures not identified via gene sequencing were (n = 2) [19,28]; author’s contact information was not available in both the cases. However, based on phenotypic methods, one study identified the isolate as *Emergomyces crescens* [19] and *Emergomyces* species in the other study [28]. Further, two studies identified *Emergomyces* species with molecular methods; however, speciation was not provided, and the authors were contacted but failed to get a response [24,37].

## Data Availability

The data presented in this study are available in the article or in Supplementary Materials.

## Supplementary Materials

The following supporting information can be downloaded at: https://www.mdpi.com/article/10.3390/jof9101039/s1, Table S1: Clinical manifestations in emergomycosis patients; Table S2: Opportunistic infections in emergomycosis patients; Table S3: Descriptions of skin lesions in emergomycosis patients; Table S4: Radiological findings in emergomycosis patients; Table S5: Antifungal treatment in the emergomycosis patients.
