# Supplementary material for: Emergomycosis, an Emerging Thermally Dimorphic Fungal Infection: A Systematic Review
Source: J Fungi (Basel). 2023 Oct 23;9(10):1039. doi: 10.3390/jof9101039 (PMC10607913; doi:10.3390/jof9101039)
Supplement: Supplementary file 1 [file jof-09-01039-s001.zip › jof-2629572-supplementary.pdf]

Table S1. Clinical manifestations in emergomycosis patients

Signs and symptoms	Total N (%)	HIV-Infected patients N (%)	HIV-uninfected patients N (%)	P value
Fever	46 (59.7)	38 (49.4)	8 (10.4)	0.403
Loss of weight	36 (46.8)	32 (41.6)	4 (5.2)	0.089
Cough	35 (45.5)	29 (37.7)	6 (7.8)	0.577
Dyspnoea	13 (16.9)	7 (9.1)	6 (7.8)	0.023*
Tachypnoea	8 (10.4)	7 (9.1)	1 (1.3)	>0.99
Night sweats	10 (13)	10 (13)	0	0.110
Haemoptysis	3 (3.9)	3 (3.9)	0	>0.99
Diarrhoea	14 (18.2)	12 (15.6)	2 (2.6)	0.721
Generalised lymphadenopathy	8 (10.4)	6 (7.8)	2 (2.6)	0.668
Hepatomegaly	6 (7.8)	6 (7.8)	0	0.334
Skin lesions	65 (84.4)	58 (75.3)	7 (9.1)	0.0001*

Note: The values in the table are expressed in N (number) and % (percentage). The percentages in the table were calculated using the total number of patients (N=77) as the denominator.

*The P values <0.05 were considered statistically significant.

Table S2. Opportunistic infections in emergomycosis patients

Infections	Total<br>N (%)	HIV-Infected patients<br>N (%)	HIV-uninfected patients<br>N (%)	P value
Opportunistic Infections	*29 (37.7)*	*26 (33.8)*	*3 (3.9)*	*0.092*
Fungal Infections	*11 (14.3)*	*10 (13.0)*	*1 (1.3)*	*0.442*
Aspergillosis	1 (1.3)	0	1 (1.3)	0.208
Candidiasis	6 (7.8)	6 (7.8)	0	0.334
Cryptococcosis	1 (1.3)	1 (1.3)	0	>0.99
Histoplasmosis	3 (3.9)	3 (3.9)	0	>0.99
Bacterial Infections	*15 (19.5)*	*13 (16.9)*	*2 (2.6)*	*0.723*
Mycobacterium tuberculosis	4 (5.2)	4 (5.2)	0	0.574
Syphilis	1 (1.3)	1 (1.3)	0	>0.99
Non-tuberculous mycobacteria	5 (6.5)	5 (6.5)	0	0.577
Bacteremia	6 (7.8)	4 (5.2)	2 (2.6)	0.599
Viral infections (other than HIV)	*9 (11.7)*	*8 (10.4)*	*1 (1.3)*	*0.675*
Parvovirus	1 (1.3)	1 (1.3)	0	>0.99
Kaposi sarcoma	1 (1.3)	1 (1.3)	0	>0.99
Herpes virus	3 (3.9)	3 (3.9)	0	>0.99
Hepatitis B and C virus	3 (3.9)	3 (3.9)	0	>0.99
Cytomegalovirus	1 (1.3)	0	1 (1.3)	0.208

Note: The values in the table are expressed in N (number) and % (percentage). The percentages in the table were calculated using the total number of patients (N=77) as the denominator.

The P values <0.05 were considered statistically significant.

Table S3. Descriptions of skin lesions in emergomycosis patients

Appearances of skin lesions	Total N (%)	HIV-Infected patients N (%)	HIV-uninfected patients N (%)	P value
Skin lesions [a]	*65 (84.4)*	*58 (75.3)*	*7 (9.1)*	*0.0001*[*]
Papules	32 (41.6)	28 (36.4)	4 (5.2)	0.162
Nodules	17 (22.1)	13 (16.9)	4 (5.2)	0.743
Crust	15 (19.5)	13 (16.9)	2 (2.6)	0.723
Ulceration	12 (15.6)	9 (11.7)	3 (3.9)	0.706
Plaques	11 (14.3)	10 (13)	1 (1.3)	0.442
Macules/Patches	11 (14.3)	11 (14.3)	0	0.107
Erythema	10 (13)	10 (13)	0	0.110
Verrucous	5 (6.5)	5 (6.5)	0	0.577
Dry Scales	4 (5.2)	4 (5.2)	0	0.574

Note: The values in the table are expressed in N (number) and % (percentage). The percentages in the table were calculated using the total number of patients (N=77) as the denominator.

[*]The P values <0.05 were considered statistically significant.

[a.] The descriptions of the skin lesions were reported in n=54 patients (HIV-infected (n=48) and HIV-uninfected (n=6)).

Table S4. Radiological findings in emergomycosis patients

Radiological findings	Total N (%)	HIV-Infected patients N (%)	HIV-uninfected patients N (%)	P value
Radiological findings of chest [a]	*58 (75.3)*	*44 (57.1)*	*14 (18.2)*	*0.330*
Lung opacities and infiltrates	46 (59.7)	32 (41.6)	14 (18.2)	0.011*
Hilar lymphadenopathy	19 (24.7)	16 (20.8)	3 (3.9)	0.747
Lung collapse	4 (5.2)	3 (3.9)	1 (1.3)	>0.99
Pleural effusion	3 (3.9)	2 (2.6)	1 (1.3)	0.508
Lung mass	3 (3.9)	1 (1.3)	2 (2.6)	0.108
Tubercular cavities	4 (5.2)	3 (3.9)	1 (1.3)	>0.99
Pericardial effusion	2 (2.6)	2 (2.6)	0	>0.99
Radiological findings of the abdomen [a]	*21 (27.3)*	*21 (27.3)*	*0*	*0.004**
Abnormal liver findings	12 (15.6)	12 (15.6)	0	0.062
Splenic Lesions	6 (7.8)	6 (7.8)	0	0.334
Abdominal lymphadenopathy	7 (9.1)	7 (9.1)	0	0.334
Abnormal Renal Findings	2 (2.6)	2 (2.6)	0	>0.99
Abdominal mass	2 (2.6)	2 (2.6)	0	>0.99

Note: The values in the table are expressed in N (number) and % (percentage). The percentages in the table were calculated using the total number of patients (N=77) as the denominator.

*The P values <0.05 were considered statistically significant.

[a.] Radiological findings in the chest and abdomen were reported in n=58 and n=21 patients, respectively. Multiple findings were documented in each patient.

Table S5. Antifungal treatment in the emergomycosis patients

Treatment	Total<br>N (%)	HIV-Infected patients<br>N (%)	HIV-uninfected patients<br>N (%)	P value
Anti-tubercular (ATT) and Anti-retroviral Therapy (ART)				
ATT for active disease	4 (5.2)	4 (5.2)	0	0.574
ATT empirical treatment	30 (39)	30 (39)	0	0.0001*
On ART during the presentation	30 (39)	30 (39)	0	0.0001*
ART newly initiated after diagnosis	23 (29.9)	23 (29.9)	0	0.002*
ART default before skin lesions	14 (18.2)	14 (18.2)	0	0.034*
Never on ART	3 (3.9)	3 (3.9)	0	>0.99
Antifungal Treatment [a]				
Patients on antifungal therapy	64 (83.1)	50 (64.9)	14 (18.2)	0.740
No antifungal treatment	9 (11.7)	8 (10.4)	1 (1.3)	0.740
Amphotericin B alone	8 (10.4)	7 (9.1)	1 (1.3)	>0.99
Triazole alone	*19 (24.7)*	*12 (15.6)*	*7 (9.1)*	*0.058*
Fluconazole alone	7 (9.1)	7 (9.1)	0	0.334
Itraconazole alone	7 (9.1)	3 (3.9)	4 (5.2)	0.031*
Posaconazole alone	1 (1.3)	0	1 (1.3)	0.208
voriconazole alone	1 (1.3)	0	1 (1.3)	0.208
Itraconazole and Fluconazole	2 (2.6)	2 (2.6)	0	>0.99
Itraconazole, Fluconazole and Posaconazole	1 (1.3)	0	1 (1.3)	0.208
Amphotericin B and azole combination therapy	*34 (44.2)*	*30 (39)*	*4 (5.2)*	*0.098*
Amphotericin B and Itraconazole	24 (31.2)	23 (29.9)	1 (1.3)	0.016*
Amphotericin B and Fluconazole	5 (6.5)	4 (5.2)	1 (1.3)	>0.99
Amphotericin B, Itraconazole and Fluconazole	4 (5.2)	3 (3.9)	1 (1.3)	>0.99
Amphotericin B, Voriconazole and Fluconazole	1 (1.3)	0	1 (1.3)	0.208
Amphotericin B, azoles and echinocandins	*3 (3.9)*	*1 (1.3)*	*2 (2.6)*	*0.108*

Amphotericin B, Posaconazole and Caspofungin	1 (1.3)	0	1 (1.3)	0.208
Amphotericin B, Voriconazole and Caspofungin	1 (1.3)	0	1 (1.3)	0.208
Amphotericin B, Itraconazole, Fluconazole and Caspofungin	1 (1.3)	1 (1.3)	0	>0.99

Note: The values in the table are expressed in N (number) and % (percentage). The percentages in the table were calculated using the total number of patients (N=77) as the denominator.

[*]The P values <0.05 were considered statistically significant.

[a.] Antifungal treatment data was available in 73 cases. Four patients did not have treatment data [10,30].
